# Trichorrhexis Nodosa in Mules

**Published:** 1899-06

**Authors:** 


TRICHORRHEXIS NODOSA IN MULES. By Falotti.—Among the mules of one of the Alps battalions Fallotti observed the following disease of the hair: On the croup, the region of the loins, and the ribs the skin was perfectly intact. The hair did not fall off here, but broke off. The latter took place especially at knotty, raised points visible to the naked eye and resting, as it were, some centimetres from the skin itself. From these bald spots arose, as if the hair had been cut off with the shears. These spots were not round, but in strips, like the markings on the zebra. In these places, after the disappearance of the trouble, dark or white hair was found, according as the animal was bay or gray.

The disease is easily communicated from animal to animal by means of currycomb and brush or harness. A microscopical examination of the hair, which had lain for some time in a 5 per cent. alkali solution, showed a ravelling or splitting up of the hair on the knotty excrescence, as if two brushes had been pushed into each other. Without coloring no microörganisms could be detected on these places, but when methylene-blue or violet was applied they appeared in great numbers, some arranged singly, some in chains, particularly on the edges of the excrescences.

Perroncito and Bosso confirmed this discovery, although Falotti himself admits that, for lack of experimental investigation, he knows nothing of the nature of this so-called disease, but is inclined to consider the microörganisms found the cause.

As a remedy Falotti found a 2 to 5 per cent. solution of sublimate to be most effective. This produced healing in about two weeks when applied every other day. He compares the trouble with human trichorrhexis nodosa, where the same condition has been found.—*Idem.*

